# Mapping the Cardiometabolic Patient Experience and Self-Care Behaviors to Inform Design, Implementation, and Persistent Use of Digital Health Care Solutions: Mixed Methods Study

**DOI:** 10.2196/43683

**Published:** 2024-01-12

**Authors:** Jan Liska, Marie Mical, Christophe Maillard, Cécile Dessapt, Europa Bendig, Daniel Mai, John D Piette, Sabina De Geest, Guillaume Fontaine

**Affiliations:** 1 Sanofi Paris France; 2 STURM und DRANG Hamburg Germany; 3 Department of Health Behavior Health Education School of Public Health University of Michigan Michigan, MI United States; 4 Institute of Nursing Science Department Public Health University of Basel Basel Switzerland; 5 Academic Center for Nursing and Midwifery Department of Public Health and Primary Care Katholieke Universiteit Leuven Leuven Belgium; 6 Ingram School of Nursing Faculty of Medicine and Health Sciences McGill University Montreal, QC Canada; 7 Centre for Clinical Epidemiology Lady Davis Institute for Medical Research Montreal, QC Canada; 8 Centre for Nursing Research Jewish General Hospital – Centre intégré universitaire de santé et de services sociaux West-Central Montreal, QC Canada; 9 Centre for Implementation Research Clinical Epidemiology Program Ottawa Hospital Research Institute Ottawa, QC Canada

**Keywords:** self-care, adherence, digital health, design, implementation, coronary, type 2 diabetes, care, patient engagement, behavior, interview, treatment, tool, digital tool, support

## Abstract

**Background:**

Cardiometabolic conditions including acute coronary syndrome (ACS) and type 2 diabetes (T2D) require comprehensive care and patient engagement in self-care behaviors, and the drivers of those behaviors at the individual and health system level are still poorly understood.

**Objective:**

We aim to gain insights into self-care behaviors of individuals with cardiometabolic conditions.

**Methods:**

A convenience sample of 98 adult patients with ACS and T2D was recruited in the United States, Germany, and Taiwan to participate in a mixed methods study using ethnographic methods. All participants completed 7-day web-based diaries tracking their level of engagement, and 48 completed 90-minute web-based semistructured interviews between February 4, 2021, and March 27, 2021, focusing on themes including moments of engagement. Qualitative analysis identified factors influencing self-care practices and a Patient Mind States Model prototype.

**Results:**

Patient reports indicate that many patients feel social pressure to adhere to treatment. Patients’ experience can be understood within 5 categories defined in terms of their degree of engagement and adherence (“ignoring,” “struggling,” “juggling,” “controlling,” and “reframing”).

**Conclusions:**

For people living with ACS and T2D, the self-care journey is defined by patterns of patient experiences, which can identify areas that tailored digital health care interventions may play a meaningful role.

## Introduction

The development of effective self-care behaviors is essential for patients living with chronic health conditions [1]. However, the definition of self-care behavior varies considerably between medical disciplines. From recent concept analyses, a broad term for self-care has been defined as “the ability to care for oneself through awareness, self-control, and self-reliance in order to achieve, maintain, or promote optimal health and well-being” [2]. Indeed, inadequate engagement in these self-care ability driving areas, suboptimal adherence to medication, and failure to enact healthy behaviors (eg, smoking cessation and physical activity) can compromise patients’ quality of life and health outcomes [1]. Self-care is particularly important for patients diagnosed with cardiometabolic conditions such as acute coronary syndrome (ACS) and type 2 diabetes (T2D) [3,4]. Despite this, rates of adherence to a care plan including but not limited to medication and lifestyle modifications remain suboptimal for this population [5-8], and a number of patient-specific factors (eg, depressive symptoms, attitudes toward management, and daily activities) that impact self-care and outcomes have been identified [9].

Despite efforts to establish the psychosocial factors underlying self-regulatory or self-management behaviors [10,11], the typical driver of such studies is to assess how these factors may impact adherence. Behavioral models have been considered within this research, such as the capability, opportunity, motivation, and behavioral model and intervention mapping; however, the volume of frameworks available and a fragmented, confusing taxonomy remain barriers to their effective use [12]. Equally, these frameworks are often generalized and applicable in a range of clinical scenarios and are not exclusive to diabetes or ACS.

This model design approach from a general perspective may explain in part why these models have had a limited impact in diabetes or cardiovascular care, where individual behavior plays a dominant role in patient outcomes [13]. Establishing a behavioral framework that is tailored for those with a cardiometabolic condition will form an important step toward understanding attitudes in clinical care and how these may innately fluctuate over time.

The determinants of effective self-care practices among people living with cardiometabolic diseases have been well-established and include self-efficacy, social support, cognitive skills, and positive attitudes [14-16]. Health care professionals (HCPs) and care systems play a central role in influencing self-care through these determinants as well as by optimizing health service delivery, addressing financial burdens, promoting engagement with technology, and encouraging community support initiatives [17,18]. As self-care determinants include both patient-level and system-level drivers, effective interventions must address both environmental factors and individual patient needs to achieve sustainable effects [19,20]. Designing these interventions requires a deep understanding of patients’ needs as well as their broader life context [21]. Patient activation–focused frameworks such as the Patient Activation Measure (PAM) or the Social, Psychological, Usage, Rational model have helped design interventions that improve medication adherence and lifestyle behaviors [22,23]. However, given ongoing changes in health systems’ use of digital patient support tools in the context of the COVID-19 pandemic, the ways in which tools such as the PAM and Social, Psychological, Usage, Rational model can best be used to support patient self-care needs further exploration. In particular, the COVID-19 pandemic has led to major shifts in clinical practice, including an increase in the use of digital health care approaches [24].

To better understand the ways in which health systems can support patients using digital support tools in the era of the pandemic, we conducted a survey of adult patients with ACS and T2D as well as their caregivers in the United States, Taiwan, and Germany. Our goal was to help establish a behavioral model based on the survey results, which would help inform how digital solutions may be utilized to improve adherence to therapy. This series of interviews and diaries, which drew on grounded theory and phenomenology, aimed to (1) provide deep human-centric insights into the behavioral dimension of the patient experience for people living with T2D or ACS and (2) identify the moments and motivational triggers in a patient’s life that have a strong impact on behavior and health outcomes.

## Methods

### Research Design

The survey consisted of 2 distinct phases, with all participants completing 7-day web-based, ethnographic diaries tracking their level of engagement in self-management and 48 participants completing web-based, semistructured interviews focused on themes including moments of engagement.

This survey used methods from the field of qualitative research, grounded theory [24] and phenomenology [25,26]. Grounded theory has been used to understand the processes through which patients manage new or chronic health problems [27], and as such, it has particular relevance to elucidating chronically ill patients’ experiences. Using grounded theory, data are collected and analyzed, and then a theory based on the resulting data was developed. The approach was designed to generate a theoretical explanation using both inductive and deductive approaches to a social phenomenon (ie, chronic disease self-care in this survey) from empirical data rather than a preconceived framework. Phenomenology is well suited to study the self-care of new or chronic health problems as it is based on the assumption that there is an essence to what people live with every day, and it aims to depict the basic structure of this experience [25]. As a qualitative research method that is particularly useful to study affective, emotional, and intense human experiences, phenomenology is a study of people’s conscious experience of their everyday life and social action [25].

### Ethical Considerations

Informed consent was obtained from all individual participants included in this survey. The research is a qualitative behavioral research survey but not a clinical study or clinical survey. This research was conducted in accordance with the organization’s intended regulations for qualitative market research studies and no personal data or sensitive information were collected or presented in this publication. Information was recorded by the investigator in such a way that the identity of the human subjects cannot be directly or indirectly ascertained. This study was exempt from the institutional review board oversight in accordance with exemption guidelines listed in the 45 Code of Federal Regulations Part 46 and the Secretary’s Advisory Committee on Human Research Protections Recommendations on benign behavioral intervention of the Health and Human Services regulation in United States[28,29]; the 2022 European Pharmaceutical Market Research Association Code of Conduct for the market research conducted in Germany [28]; and Article 5 of the Human Subjects Research Act of the Ministry of Health and Welfare, Republic of China (Taiwan), and the “Exempt Review Categories for Human Research” announced by Department of Health, Taiwan [29,30].

### Sampling Methods

Patients diagnosed with either ACS or T2D and cardiovascular comorbidities were identified and recruited in 3 countries: the United States, Germany, and Taiwan. These countries were selected based upon the Hofstede 6D model of cultural dimensions. The 3 countries were noted for their diversity of cultural dimensions that may influence and nurture patient behavior while holding similar maturity of health care systems.

We aimed to recruit equal proportions of men and women as well as patients with a variety in types of health insurance. Potential patients were members of an ongoing research panel who have agreed to be approached for research studies and have provided basic demographic and health status information. Patients with ACS and T2D were screened for eligibility using elements for ACS and T2D patient profiles, as well as survey-specific data regarding demographics, digital behavior, and personality profiles. As part of the screening process, patients were asked if they use any technology, either app or device, to manage their condition. All patients screened were interested and open to the idea of digital health care (ie, the use of digital tools to support tracking or managing measurements used to help manage their condition).

Screening was conducted by an external recruiting agency who preselected relevant candidates out of their patient panel. In total, 32 participants per country were selected out of approximately 48 recommended profiles per country from an external recruiting agency. An appropriate mix of people was selected by the research lead, striving for maximum variation in the sample among those who fulfill the sampling criteria.

Our goal was to recruit 16 patients with ACS from each country, including 8 patients who had undergone an invasive procedure (eg, percutaneous coronary intervention and coronary artery bypass graft). We also sought to recruit roughly equal numbers of patients with ACS who were aged 40-50 years and 50-70 years. All patients had to be eligible for enrollment in a cardiac rehabilitation program (whether this was digital or analog was recorded); approximately 60% (10/16) should have attended rehabilitation, while 40% (6/16) should not. A focus was placed on those with higher severity levels for their condition and those with progressed treatment regimens. Eligibility for enrollment in a cardiac rehabilitation program was determined as a proxy for ACS severity levels and an indicator of having undergone a medical procedure. In recruiting patients with ACS, our goal was to represent a diversity of family situations (single, married, and with or without children) and a range of experiences using self-care technology for managing cardiovascular conditions. Finally, we sought to recruit 10-16 caregivers of patient with ACS to assist in setting up patient support with the diaries, 8 of whom would also serve as interview participants.

Our goal was to recruit 16 patients with T2D from each country, with equal numbers having less severe (eg, hypertension) and more severe (eg, heart arrythmia and heart failure) comorbidities. These groups were each further equally split into those on oral antidiabetic drugs and those who had initiated basal insulin in the past 12 months (18-24 months if recruitment was difficult). All participants in the T2D sample were aged 40-70 years, with the group being split equally between those who currently use health care technology (an app or device) and those who are considered lapsed users of health technology. Within the screening process, patients with T2D were asked if they use any technology, either an app or device, to manage their disease. Lapsed users confirmed that they used technology to manage their condition but no longer do so.

Both the ACS and T2D samples were split into equal-sized groups of patients deemed to be fully adherent with medical advice and treatment and those considered either partly adherent or nonadherent. This was based on the assumption that the behavioral reasons for partial and nonadherence were similar. Adherence levels were determined based on patients’ self-reported assessment during the respondent recruitment process. Patients were asked a range of multiple-choice and open-ended questions, the results of which were used to quantitatively determine their adherence level. For instance, patients were asked to what extent they agree with the statement, “I am confident that I can follow through on medical recommendations my health care provider makes, such as changing my diet or regular exercise;” with which they could strongly agree, somewhat agree, somewhat disagree, or strongly disagree.

### Data Collection

To increase internal validity of the survey through triangulation, 2 methods of data collection common in grounded theory and phenomenology research were used [26]. First, patients were asked to complete web-based diaries for 7 days, in which they recorded information including their daily experience of engagement with notable life events, health, and conditions (eg, photos and video) for acute versus chronic; a mind map of helpers (people, tools, and institutions); and observations of self-concept versus reality (projections and narratives around the self-care experience).

The diaries consisted of 5 chapters, with each one completed once within 7 days. Each chapter was completed in consecutive order: (1) “Me and My Body,” (2) “My Story,” (3) “My Day-to-Day Life with My Condition,” (4) “My Daily Health Regimen,” and (5) “My Helpful Tools and Resources.” Daily diary exercise was recorded based on open-ended questions with private responses recorded via video, audio, photographs, a map, or written statement.

Specifically, respondents were asked to answer a range of open-ended questions over the course of 5 days. They were structured in six thematic buckets: (1) personal background, (2) me and my body, (3) daily health regimen, (4) day-to-day life with my condition, (5) helpful tools and resources, and (6) my health story. In topic 2, we asked, for instance, “Please recall particularly pleasurable and positive moments during the day when you felt happy, in control, proud of yourself, content with regards to a) general state of mind, b) relating to health, and c) relating to your condition.”

For the interviews, participants were screened by an external recruiting agency, relying on their patient panels, and an appropriate mix of people was selected by the research lead, striving for maximum variation in the sample among those who fulfill the sampling criteria. In the ACS sample, patients and their caregivers took part in dyad interviews. In the T2D sample, interviews were carried out on a one-to-one basis between interviewers and patients. These interviews aimed to capture key moments of engagement to gain an understanding of levers of change or opportunities; elucidate the role or influence of the caregiver; and explore a prototype of the patient experience map produced from patient diary content. The “SturmundDrang” team of cultural researchers and anthropologists created the interview guide in an iterative approach. This guide provided information on field research preparations. Designed as a rough framework of topics and themes to explore, this document offered guidance for the ethnographic video interviews. The same discussion guide was used across all countries, providing open-ended questions and sufficient room for exploration in different cultural contexts. The respective researcher could modify the guides in each country, if deemed necessary.

### Data Analysis

Data were analyzed using a constant comparative method [31], which involves comparing 1 segment of data in an interview with another segment of data to determine similarities and differences [26]. The diary data was treated as 1 hermeneutic unit that was then qualitatively analyzed in direct comparison with the interview data. The interview data formed a second hermeneutic unit. Diaries and interviews were conducted within 2 weeks, allowing the researchers to pursue a close comparative approach. These 2 hermeneutic units were then compiled in a comprehensive raw field note document for each market, outlining the total qualitative data set that could then be analyzed.

Analysis was guided by the principles of horizontalization, where all data are treated with equal weight, and phenomenological reduction, which is the process of continually returning to the essence of the experience to derive the inner structure (of disease self-care) in and of itself [26]. We grouped data together on similar dimensions of (1) patient modes of engagement and experience domains, (2) the ongoing process of gaining self-care expertise, and (3) patient mind states regarding disease self-care. Furthermore, we identified beliefs and biases, drivers of engagement and challenges to disease self-care.

In particular, interviews were analyzed to identify emerging themes through established social-scientific methods of data gathering, including writing semistructured fieldnotes [32], qualitative data analysis [33], and ethnographic insights building [34]. To establish key patient mind states, the data were clustered using spatial clustering, iterative loops, and narrative listening. Spatial clustering of codes and signals was used to group themes into a figure of overarching mind states to provide a framework for qualitative interpretation and a hypothesis. Following data collection, the resulting map was refined through iterative loops, whereby the data and theory were examined and re-examined by a team of 3 researchers; with each loop, the number of clusters and mind states was adapted and refined. During the final iteration loop, the mind states and their names were co-designed with the patients to ensure a patient-centric outcome. Narrative listening was used throughout, with patients describing and naming the chapters of their journey to allow for patient-led clustering and refining of the mind states [35].

## Results

### Sample Characteristics

In total, 98 patients with ACS or T2D and cardiovascular comorbidities were recruited, 32 from the United States and 33 from each of Germany and Taiwan. All 98 patients who were recruited completed the survey. All patients completed the 7-day ethnographic diaries (Germany or United States: February 4-11, 2021; Taiwan: February 17-24, 2021). Patients spent approximately 5 hours to complete the research questions. In total, 48 web-based interviews were conducted (Germany or United States: March 3-15, 2021; Taiwan: March 22-27, 2021), 24 with patients with T2D and 24 with patients with ACS and their caregivers. Respondents were selected for further interviews based on the depth of patient journey detail.

### Survey Results

#### Overview

From the survey conducted through interviews and diaries, a number of theoretical themes were identified.

#### Insights Into the Behavioral Dimension of the Patient Experience

Interviews with patients living with T2D indicated that the disease continues to extract a heavy emotional toll. Participants commented: “The nurse made me feel like some pig, that all I do is sit around and eat and be obese”; “I used to eat gourmet food anywhere, anytime, and now all I have left is pay attention to controlling my diet”; “If I were to buy something to eat – I almost never do – then I would have to take everything apart and weight the ingredients individually; I can then calculated what units I need to inject afterwards”; and “I thought I’d just take a pill and everything will be ok, so I didn’t take it as seriously as I should.”

These patient insights highlighted how T2D is still a chronic condition that complicates every aspect of one’s life. Overarching themes from these interviews indicate that the condition is widely perceived to be a self-inflicted lifestyle disease; patients experience feelings of stigma, shame, self-blame, and a need to justify their lifestyle choices to acquaintances and HCPs. The general experience is underscored by patient sacrifices and unsolicited social pressures to adhere to treatment regimens; generating a depressive, restrictive atmosphere rather than the positive outlook that could help patients pursue a better quality of life. The need for constant monitoring and tracking of the body increases adherence pressure and creates a heightened focus on hemoglobin A_1c_ levels. Patients can also initially find diabetes easy to underestimate and ignore, adopting an acute rather than chronic mindset that makes it difficult to take ownership in the long term.

Acute events often are perceived by patients as inevitable consequences of living with chronic conditions. For example, 1 patient with ACS commented: “I learned that even without heart disease, unhealthy habits as far as eating and exercise can lead to stroke and heart failure. This really opened my eyes. At that point my life changed dramatically. I changed the way I shopped, I started eating healthier meals, and I began exercising regularly.” However, acute conditions may have a longer-term impact on social behaviors. ACS can lower social and career performance expectations; patients may feel forced out of the “rat race” by their condition or voluntarily take steps to reduce work-related burdens and pressures, as indicated by 1 patient who commented: “I see people around me going to work and realize how much my health prohibits me from working.”

Based on these moments and experiences, patients may feel that rehabilitation is a challenge and may not fully comprehend or appreciate the potential benefits. Once the advantages of rehabilitation are clearly communicated, the offering is compelling to patients if logistical barriers are no issue.

**Figure 1:**
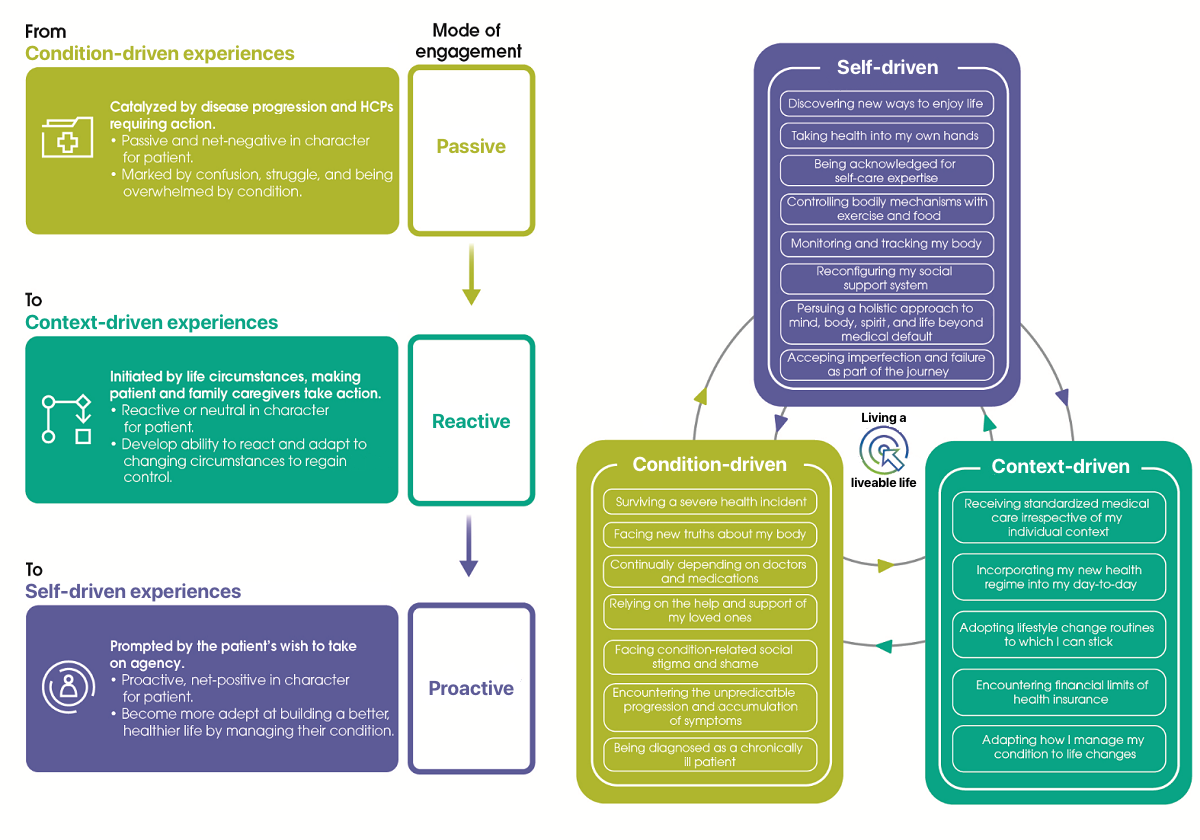
Patient modes of engagement and experience domains. HCP: health care professional.

Participants also described the gradual acquisition of self-care expertise as a journey, which can support them in working toward better health. They also see this journey as influenced by universal health-related experiences, where a patient undergoes a cognitive or emotional change regarding their health engagement and self-care. Although each patient journey is unique, we identified 3 modes of engagement that determine the engagement and behavioral patterns in self-care (Figure 1). Underlying these modes are clusters of experiences called “experience domains.” At different points in the patient journey, health-related experiences from any domain can affect the patient’s mode of engagement, in a process of fluid exchange and even overlap between domains.

**Figure 2:**
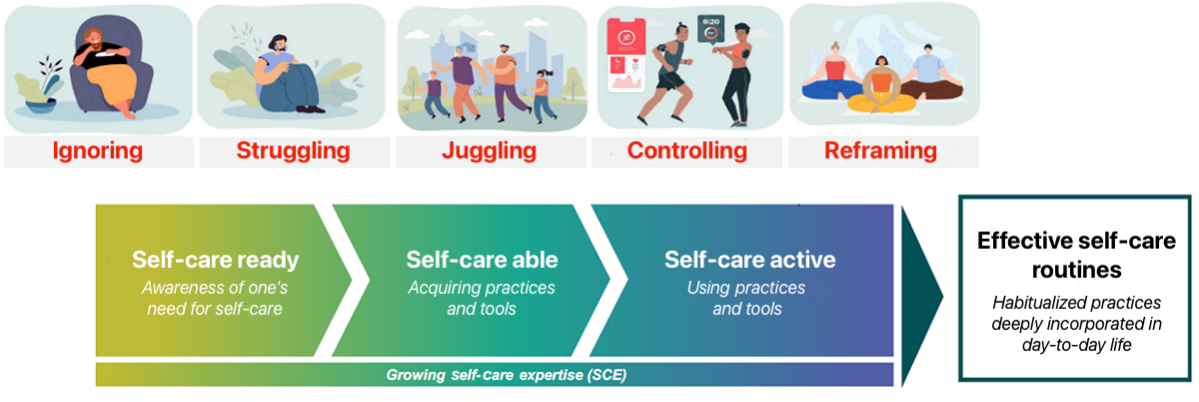
The ongoing process of gaining self-care expertise.

Self-care can be seen as an ongoing process of gaining expertise on how to deeply incorporate management routines into patients’ day-to-day lives (Figure 2). Over the years of encountering the full breadth of health care–related experiences, patients work out the best ways to manage their conditions in the context of their individual lives. This process takes patients from awareness of the need for self-care, through acquiring the necessary practices and tools to learning how to use them successfully. A set of applicable self-care practices are developed, which become habits in the form of routines. Highly engaged patients eventually become informal self-care experts on their own body and health.

Interviews and patient log data indicate that a patient’s willingness to engage in self-care and gain expertise may be shaped by the cultural context in which they reside. Some of the participants in the United States expressed a growing mistrust of medical expertise and institutions, with a lack of medical insurance also disrupting the continuity of care. For example, patients from the United States shared: “Never trust someone just because they have a medical degree.” And “I’m on Medicare and Medicaid, I’m disabled, cannot work and now I’m getting paid back for the things I used to say because the clinic won’t cover it.” Effective self-care may also present challenges in countries such as Germany, where many health care systems rely primarily on paper-based clinical records, owing to historical and ongoing data privacy concerns. This impacts the patient experience. A man with T2D from Germany shared: “I am fortunate that my two doctors are located in one facility and can therefore coordinate closely. All the information [about my treatment] is centrally stored and can be viewed at the facility at any time.” Pseudoscientific or alternative approaches to health care, such as homeopathic or organic products as well as spiritual practice, also remain prevalent in Germany and Taiwan; however, participants in Taiwan expressed a high level of trust in HCPs’ expertise and authority, with 1 participant commenting: “I’ll strictly follow the doctor’s advice as best as possible.”

These differing experiences across countries was a common theme during the survey. Although, patients in the United States expressed a mistrust toward institutions, those surveyed were enthusiastic about trying new digital health solutions. However, this attitude was not reflected in Germany, with privacy concerns limiting the uptake of digital solutions. Many patients remain cautious around such technologies. Further, 1 patient with T2D in Germany stated: “I don’t know what I would want [for a digital solution]...and what is possible privacy wise.” Both US and German attitudes toward digital health care stand in contrast with the opinions of those patients in Taiwan. There has been an increasing adoption of digital solutions among those with T2D in Taiwan, with many perceiving such innovations as convenient and readily integrable.

#### Theorization of Patient Engagement: the Patient Mind States Model (PMM)

**Figure 3:**
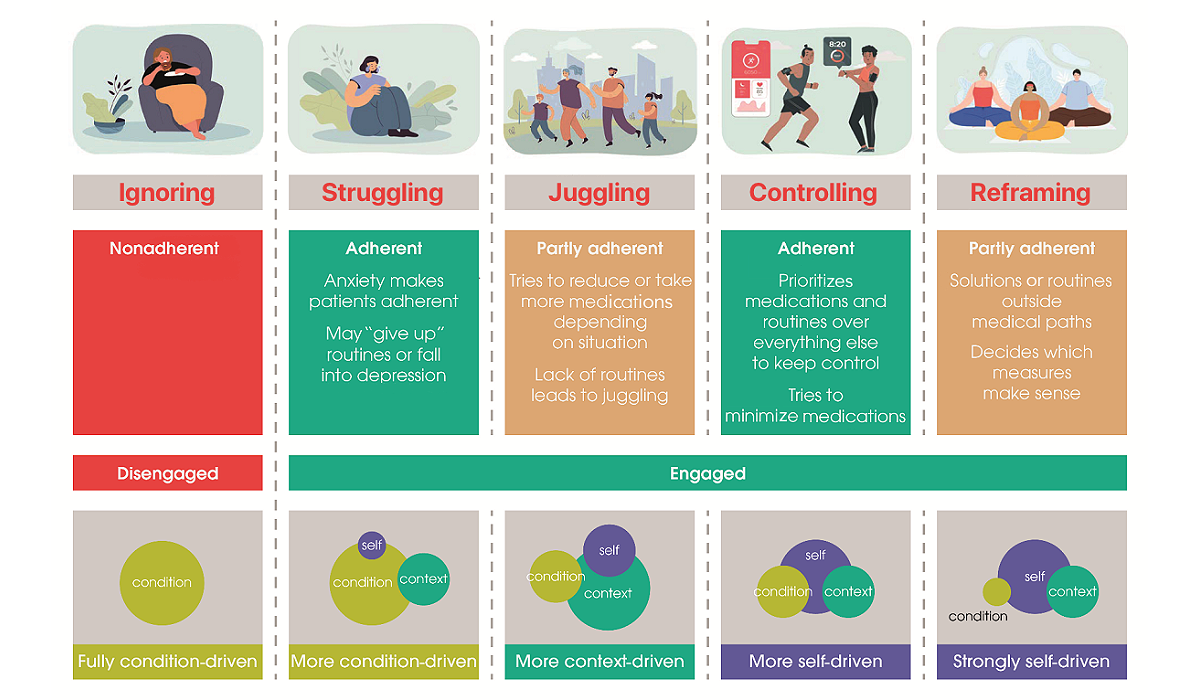
The Patient Mind States Model (PMM)—this model contains patient mind states regarding disease self-care identified in patients with chronic cardiometabolic disease.

Based on analyses of the survey through patient interviews, we identified 5 patient mind states according to degree of patient engagement, adherence, and the experience domains that drive engagement (namely, the condition-, context-, and self-driven experiences; Figure 3). These mindsets comprise what we have called the Patient Mind States Model (PMM), which articulates these 5 mind states regarding disease self-care. A mind state is defined as a patient’s mental and emotional attitude toward self-care; these states are not related to age, gender, sociodemographic criteria, or culture of persons living with cardiometabolic conditions. The mind state has a large influence on a patient’s receptivity toward support and their ability to develop more healthy behavior. Patients’ mind states are not constant; shifts in mind state can be driven by external forces such as seasonal cycles and life changes. Self-care engagement and maturity of patients with chronic conditions change with their mindset. The 5 mind states identified are the following:

- Ignoring: patients carry on with life as it was prior to their diagnosis; depending on their culture and the extent to which they are stuck in an acute mindset, they may believe that a drug can fix the problem. For example, a 68-year-old woman with T2D from Germany commented: “I don’t really feel sick, because I don’t notice anything. I don’t have a different life in terms of ‘before’ or ‘after’ the diagnosis. Actually, I ignore the disease.”
- Struggling: patients feel overwhelmed and anxious; in desperately trying to make sense of what is happening, they can be paralyzed and oscillate between desperation and overambition. A 59-year-old woman with ACS from Germany shared: “I sleep very badly, have fears about my health. I feel completely overwhelmed and don’t know how to get out of this dilemma.”
- Juggling: people want to focus on positive aspects of life, which are viewed as part of the healing process; they see themselves as trying to find a balance between their own wants and the demands of their condition. One 64-year-old man with ACS from Germany commented: “Life should still be fun, you have to continue to participate in it, even if you are sick...I do not want to miss on the taste of a beer, a glass of wine or a good meal.”
- Controlling: patients have high familiarity with the effects of food and exercise on their body; they constantly learn more about their condition and have a high use of tools to maintain control and promote a feeling of self-reliance. As noted by a 63-year-old man with T2D from the Unites States: “Being in touch with my body, it has an equal ‘voice’ in determining what is best for me. This helps me to maintain an aggressive mindset and to immediately determine any abnormalities I might be experiencing and take appropriate action.”
- Reframing: people have learned to control their disease and focus on achieving life goals; they use organic methods and vitamins or supplements combined with physical exercise, breathing techniques and meditation to reduce stress. A 61-year-old man with T2D indicated: “Just because someone has a Medical degree doesn’t mean they don’t make mistakes, listen to their advice, but also do some research for yourself. Never blindly follow anyone or anything but find your own ways.”

These mind states may share common traits through their drivers for engagement, as detailed in Figure 1. Through this survey, each mind state is not exclusively self-, context-, or condition-driven, with each having a predominant motivation.

## Discussion

### Principal Findings

Each mind state in the PMM is associated with specific beliefs and challenges and is susceptible to particular drivers of engagement that can be used to propel effective self-care behavior (Multimedia Appendix 1). In the ignoring mind state, these drivers of engagement include a fear of mortality and sense of urgency, social pressure from loved ones, and relatable role models who defy the stigma. As for the “why me” frame of mind of the struggling mind state, affirmative feedback from HCPs, realistic prognosis that shows possible points for medical intervention, and a sense of urgency that prioritizes the disease serve as drivers of engagement. In the juggling mind state, a mind state that focuses on the positive aspects of life as part of the healing process, clear images of cause and effect, gratification through joyful activities, and companionship with peer patients on their disease journey drive engagement. Moreover, people in the controlling mind state are driven to engage through curiosity for, and excitement about, innovation and what is novel; recognition of progress and being awarded for success; and having a sense of medical expertise. When patients are in the reframing mind state, drivers of engagement include an awareness that certain rules are malleable, deep trust in the individual’s own capabilities, and joy of helping peer sufferers. These emotional and social drivers of engagement across each mind state highlight the need for a holistic approach beyond the current physiological and intellectual drivers of engagement in health care, from gratification through device use to feelings of self-efficacy via immediate feedback and to encourage patient self-care behaviors.

Partitioning a person’s mind state into different stages is not a recent concept, with frameworks such as the transtheoretical model (TTM) examining different behavioral states that evolve over time [36]. The TTM consists of several stages of change and different behavioral processes that drive people to transition to a different state (eg, contemplation, determination, and action). However, the underlying assumption behind this model that decision-making is linear and unidirectional does not recognize how people may move back and forth between such states over their lifetime, further deviating from expected behavior change trajectory by exponentially changing social and technological context. The PMM described in this survey allows us to nuance further TTM by its nonlinear nature where patient mind states do not necessarily occur in sequential fashion and may even coexist.

It should also be noted that existing behavioral models are used as tools to support clinical decision-making. In diabetes care, the PAM has been leveraged to predict the potential course of outcomes and how underlying social factors may contribute to activation levels. Mean scores obtained from various PAM instruments, such as PAM-13, may offer a concise summary of a person’s knowledge, skills, and motivation. However, the assessment of drivers underlying activation levels and broader consideration of patient context and its evolution are often decoupled from a PAM assessment. In a recent study on patient activation in individuals with T2D, generic health status topics, distress, and social support were all assessed in questionnaires separate from the primary PAM survey [37]. The PMM may offer complementary perspective for assessment of the context for an individual’s mind state through 1 centralized survey and establish the patient modes of engagement and wider experiences, as detailed in Figure 1. Further studies will be required to elucidate how the PMM could form the basis of a behavioral tool in practice.

### Opportunities for Future-Focused Digital Solutions

Digital health care has an important role in chronic patient journey. However, many digital patient support programs may suboptimally tailor and target their support based on these important differences, in part because of a lack of data integration across platforms. Guidance on the day-to-day implementation of digital solutions is lacking, so patients often achieve success through trial and error.

Given a patients’ potential frustration with digital tools, engendering a level of comfort and trust in the technology is an important step to effective self-care. Along with privacy concerns, evidence for digital solutions remains as key challenges in establishing digital health as a viable solution for patient self-care [38-40].

Each patient has a unique experience in self-care, with the previous discussion outlining how we can understand the changing mind state of these individuals and how digital solutions may proffer opportunities to improve self-care.

For chronic care digital interventions, our findings suggest that it is important to tailor support to a patient’s mind state, with personal drivers of engagement potentially leading to optimized patient outcomes, adherence, and self-care expertise. Guiding patients throughout their individual health journey to a life worth living is critical and should be based on the individual, attainable life goals, and intelligently balanced compromises that undergo constant revision in the ever-changing context.

These dynamic patient experience mapping refined with help of the PMM may form a more optimal basis in which to effectively integrate digital solutions that enable and support disease self-care, while considering more holistically the context of those living with cardiometabolic conditions. These insights also may warrant further studies in the field of patient adherence and sustainable behavioral change. It will be of interest to further investigate the underlying motivations behind a change in patient mind state and how digital health care may help move individuals from “struggling” to “controlling” mind states, for instance, and effectively ignite intrinsic patient motivation drivers.

These initial data may form a basis of future studies through the validation and refinement of the PMM and the relationship between patient mind states and chronic disease self-care. In particular, future research should clarify patient self-care behaviors and attitudes toward specific digital health care interventions as a critical part of digital intervention design and development processes and verify that the user experience of participants with these 2 conditions in these 3 countries is consistent with patients in other contexts. Equally, it will be critical to understand more deeply the levers of progressive self-care expertise acquisition and use by patients.

The limitations of this survey include its geographic profile (only 3 countries) and the limited sample size of the patient populations (owing to the specific inclusion criteria). Participants who completed the survey may not be representative of the general patient population, as the survey was conducted via the web and patients participated on a voluntary basis. This approach may have introduced a selection bias, such that only the most motivated or educated patients were included. The educational needs of a representative patient population may have therefore been underestimated. Further, the PMM has been generated based on the inputs from participants who were managing ACS and T2D and has not included people living with cardiovascular chronic conditions alone or type 1 diabetes. This may result in that the PMM may not fully embrace the whole spectrum of cardiometabolic patient profiles. The focus on the social aspect of survey respondents (role of caregivers in building patient’s self-care motivation) has not been sufficient to frame more distinctly in the PMM. However, this survey had reflected the real-life experiences of patients in different clinical and geographic settings.

### Conclusions

Any single journey with an acute or chronic condition is consistently shaped by moments, as well as motivational triggers, which may impact a patient’s growing expertise in self-care. Through this behavioral science survey, a heuristically useful framework has been defined on the underlying nature of how patients engage with self-care, which requires further testing and adaptations. Patients will gradually gain expertise in self-care before acquiring more confidence in proactively using a range of practices and tools. However, this path may be shaped by the patient’s mind state, which will impact treatment adherence and their willingness to engage in self-care practices.

For digital health care solutions to be fully integrated into the patient care journey, it is important to understand how such tools should be tailored to a patient’s mind state and how these states may shift when digital solutions are adopted. It will also be important to understand that such solutions may adapt according to changes in patient mind states.

## Appendix

Multimedia Appendix 1 Key features of different patient mind states.

## Abbreviations

ACS: acute coronary syndrome
HCP: health care professional
PAM: Patient Activation Measure
PMM: Patient Mind States Model
T2D: type 2 diabetes
TTM: transtheoretical model

## Data Availability

Qualified researchers may request access to documents related to this survey. Further details on Sanofi’s data sharing criteria, eligible studies, and process for requesting access can be found online. This survey was funded by Sanofi.

## References

[1] de Geest Sabina, Sabaté Eduardo (2003). Adherence to long-term therapies: evidence for action. Eur J Cardiovasc Nurs.

[2] Martínez N, Connelly CD, Pérez A, Calero P (2021). Self-care: a concept analysis. Int J Nurs Sci.

[3] Heisler M (2007). Overview of peer support models to improve diabetes self-management and clinical outcomes. Diabetes Spectr.

[4] Kourbelis C, Franzon J, Foote J, Brown A, Daniel M, Coffee NT, Newman P, Nicholls S, Clark RA (2018). Effectiveness of discharge education on outcomes in acute coronary syndrome patients: a systematic review protocol. JBI Database System Rev Implement Rep.

[5] Twardella D, Merx H, Hahmann H, Wüsten B, Rothenbacher D, Brenner H (2006). Long term adherence to dietary recommendations after inpatient rehabilitation: prospective follow up study of patients with coronary heart disease. Heart.

[6] Iglay K, Cartier SE, Rosen VM, Zarotsky V, Rajpathak SN, Radican L, Tunceli K (2015). Meta-analysis of studies examining medication adherence, persistence, and discontinuation of oral antihyperglycemic agents in type 2 diabetes. Curr Med Res Opin.

[7] Elliott AJ, Harris F, Laird SG (2016). Patients' beliefs on the impediments to good diabetes control: a mixed methods study of patients in general practice. Br J Gen Pract.

[8] Desta L, Khedri M, Jernberg T, Andell P, Mohammad MA, Hofman-Bang C, Erlinge D, Spaak J, Persson H (2021). Adherence to beta-blockers and long-term risk of heart failure and mortality after a myocardial infarction. ESC Heart Fail.

[9] Bauer AM, Parker MM, Moffet HH, Schillinger D, Adler NE, Adams AS, Schmittdiel JA, Katon WJ, Karter AJ (2017). Depressive symptoms and adherence to cardiometabolic therapies across phases of treatment among adults with diabetes: the Diabetes Study of Northern California (DISTANCE). Patient Prefer Adherence.

[10] Gonzalez JS, Tanenbaum ML, Commissariat PV (2016). Psychosocial factors in medication adherence and diabetes self-management: implications for research and practice. Am Psychol.

[11] Lambrinou E, Hansen TB, Beulens JW (2019). Lifestyle factors, self-management and patient empowerment in diabetes care. Eur J Prev Cardiol.

[12] McSharry J, Byrne M, Casey B, Dinneen SF, Fredrix M, Hynes L, Lake AJ, Morrissey E (2020). Behaviour change in diabetes: behavioural science advancements to support the use of theory. Diabet Med.

[13] Hill-Briggs F, Adler NE, Berkowitz SA, Chin MH, Gary-Webb TL, Navas-Acien A, Thornton PL, Haire-Joshu D (2021). Social determinants of health and diabetes: a scientific review. Diabetes Care.

[14] King DK, Glasgow RE, Toobert DJ, Strycker LA, Estabrooks PA, Osuna D, Faber AJ (2010). Self-efficacy, problem solving, and social-environmental support are associated with diabetes self-management behaviors. Diabetes Care.

[15] Shin ES, Hwang SY, Jeong MH, Lee ES (2013). Relationships of factors affecting self-care compliance in acute coronary syndrome patients following percutaneous coronary intervention. Asian Nurs Res (Korean Soc Nurs Sci).

[16] Karimy M, Koohestani HR, Araban M (2018). The association between attitude, self-efficacy, and social support and adherence to diabetes self-care behavior. Diabetol Metab Syndr.

[17] Adu MD, Malabu UH, Malau-Aduli AEO, Malau-Aduli BS (2019). Enablers and barriers to effective diabetes self-management: a multi-national investigation. PLoS One.

[18] de Man J, Aweko J, Daivadanam M, Alvesson HM, Delobelle P, Mayega RW, Östenson CG, Kirunda B, Kasujja FX, Guwattude D, Puoane T, Sanders D, Peterson S, Tomson G, Sundberg CJ, Absetz P, van Olmen J (2019). Diabetes self-management in three different income settings: cross-learning of barriers and opportunities. PLoS One.

[19] Miller WR, Lasiter S, Ellis RB, Buelow JM (2015). Chronic disease self-management: a hybrid concept analysis. Nurs Outlook.

[20] van de Velde D, de Zutter F, Satink T, Costa U, Janquart S, Senn D, de Vriendt P (2019). Delineating the concept of self-management in chronic conditions: a concept analysis. BMJ Open.

[21] Moore G, Campbell M, Copeland L, Craig P, Movsisyan A, Hoddinott P, Littlecott H, O'Cathain A, Pfadenhauer L, Rehfuess E, Segrott J, Hawe P, Kee F, Couturiaux D, Hallingberg B, Evans R (2021). Adapting interventions to new contexts-the ADAPT guidance. BMJ.

[22] Hibbard JH, Stockard J, Mahoney ER, Tusler M (2004). Development of the Patient Activation Measure (PAM): conceptualizing and measuring activation in patients and consumers. Health Serv Res.

[23] Dolgin K (2020). The SPUR model: a framework for considering patient behavior. Patient Prefer Adherence.

[24] Farooqi AT, Snoek FJ, Khunti K (2021). Management of chronic cardiometabolic conditions and mental health during COVID-19. Prim Care Diabetes.

[25] Merriam SB (2015). Qualitative Research: A Guide to Design and Implementation.

[26] Kaun A (2010). Open-ended online diaries: capturing life as it is narrated. Int J Qual Methods.

[27] Jacelon CS, O'Dell KK (2005). Case and grounded theory as qualitative research methods. Urol Nurs.

[28] (2022). Code of Conduct 2022. EPHMRA.

[29] National Development Council (2012). Department of health, executive Yuan notice is hereby given, for the promulgation of "Exempt review categories for human research" (promulgation becomes effective from 5th, July 2012). The Executive Yuan Gazette Online.

[30] Chiu YL, Tsai CC, Liang JC (2022). Laypeople's online health information search strategies and use for health-related problems: cross-sectional survey. J Med Internet Res.

[31] Glaser BG, Strauss AL (1967). The Discovery of Grounded Theory: Strategies for Qualitative Research.

[32] Emerson RM, Fretz RI, Shaw LL, Atkinson P (2001). Participant observation and fieldnotes. Handbook of Ethnography.

[33] Kelle U, Kluge S (2010). Vom Einzelfall zum Typus: Fallvergleich und Fallkontrastierung in der qualitativen Sozialforschung 2nd Edition.

[34] Brewer JD, Cassell C, Symon G (2004). Ethnography. Essential Guide to Qualitative Methods in Organizational Research.

[35] Anderson C, Kirkpatrick S (2016). Narrative interviewing. Int J Clin Pharm.

[36] Prochaska JO, Prochaska JM, Rippe JM (2019). Transtheoretical model. Lifestyle Medicine, Third Edition.

[37] van Vugt HA, Boels AM, de Weerdt I, de Koning EJ, Rutten GE (2019). Patient activation in individuals with type 2 diabetes mellitus: associated factors and the role of insulin. Patient Prefer Adherence.

[38] Iyengar V, Wolf A, Brown A, Close K (2016). Challenges in diabetes care: can digital health help address them?. Clin Diabetes.

[39] Klonoff DC, Kerr D (2018). Overcoming barriers to adoption of digital health tools for diabetes. J Diabetes Sci Technol.

[40] Alexander Fleming G, Petrie JR, Bergenstal RM, Holl RW, Peters AL, Heinemann L (2020). Diabetes digital app technology: benefits, challenges, and recommendations. a consensus report by the European Association for the Study of Diabetes (EASD) and the American Diabetes Association (ADA) Diabetes Technology Working Group. Diabetologia.

