# Supplementary material for: Mapping the Cardiometabolic Patient Experience and Self-Care Behaviors to Inform Design, Implementation, and Persistent Use of Digital Health Care Solutions: Mixed Methods Study
Source: JMIR Form Res. 2024 Jan 12;8:e43683. doi: 10.2196/43683 (PMC10818241; doi:10.2196/43683)
Supplement: Multimedia Appendix 1 [file formative_v8i1e43683_app1.docx]

**Multimedia Appendix 1.** Key features of different patient mind states.

|  | **IGNORING** | **STRUGGLING** | **JUGGLING** | **CONTROLLING** | **REFRAMING** |
| --- | --- | --- | --- | --- | --- |
| **BELIEFS/BIASES** | - Illusion of invulnerability – belief of being immortal - Confirmation bias – believe things are/will be as they were - Status quo bias – believe they are too stressed/busy to change - Premature closure – think that small interventions will do | - Reduce stress to commence self-care – mental fatigue and stress hinder self-care activities - Be perfect, then take a break – start with unrealistic goals - Dependency on doctors/medication to feel safe - Self-blame, feel failure and shame | - Joy is a healer – power of a positive mindset - Life is about taking part – make compromises to enable social participation - Wisdom is in people and practices, less in technology and maths - Guilt-pleasure imbalance – cheating produces positive experiences, but also guilt | - Determination and performance are key - Unbiased and objective information equals the terms with HCPs - Understanding is key to betterment - Internalizing reactions and monitoring puts me back in control | - Form stories based on symptom experience - Spend time doing own research on medical websites - Challenge medical expertise and keep searching - Missionary transformation – proud of own self-care maturity, want to teach others |
| **DRIVERS OF ENGAGEMENT** | - Fear of mortality and sense of urgency - Convincing scare tactics employed by HCP - Social pressures, criticisms from loved ones - Relatable role models who defy stigma - Medical conversations in accessible language - Positive images of self, living with chronic condition | - Sense of urgency to prioritize disease - Positive images of self, living with chronic condition - Empathetic bedside manner and affirmative feedback from HCPs - Medical conversations in accessible language - Realistic prognosis showing points for medical intervention - Advice turning ideas into implementable actions - Positive vision for family - Financial security | - Sense of urgency to prioritize disease - Clear images of cause and effect - Advice turning ideas into implementable actions - Feeling accountable to someone who checks up on them - Gratification through joyful activities - Companionship with patient peers on disease journey - Trust in own self-care abilities - Reminders and alarms to assist memory | - Feeling of own self-efficacy through immediate feedback on effects of actions - Excitement about innovation and novelty - Recognize progress and be rewarded for success - Sense of medical expertise - Feeling of communicating on equal terms with HCPs - Feeling of receiving tailored medical care - Peer acknowledgment of self-care expertise - Comparison or competition with self and/or peers | - Awareness that some rules are malleable - Deep trust in own capabilities - Positive images of self, living with chronic condition - Excitement about innovation and novelty - Relatable role models who mastered challenges or found new ways - Feeling of communicating on equal terms with HCPs - Joy of helping peers - Faith and hope in possibility of another solution out there to fit them |
| **CHALLENGES** | - Fear of being confronted with own mortality - Understanding full implications of diagnosis - Allowing condition to become top-of-mind - Being stigmatized and lectured by others | - Making sense of symptoms and bodily changes - Isolating from public situations to avoid being the odd one out - Gaining a clear, positive view of the future - Overwhelming feelings of depression and anxiety - Unable to meet HCP expectations to comply | - Balancing self-care practices with other life obligations - Insufficient motivation to give up unhealthy behaviors - Picking up new practices and turning them into routines - Having to cut back in social situations | - Fulfilling HCP expectations of being fully compliant model patient - Being taken seriously by HCPs as amateur expert and having voice in treatment plan - Maintaining routines regardless of changing circumstances | - Finding trustworthy HCPs who respect and work with unconventional approaches - Bringing pharma skepticism and reliance on established medicine together - Receiving acknowledgement from and being listened to by, other patients |
